# Accurate breast cancer intrinsic subtyping using DNA-only biomarkers with applications to multi-cohorts

**DOI:** 10.1093/bib/bbaf631.060

**Published:** 2025-12-12

**Authors:** Xintong Chang, Linping Wang, Hongyu Duan, Li C Xia

**Affiliations:** Department of Statistics and Financial Mathematics, School of Mathematics, South China University of Technology, Guangzhou 510000, Guangdong, China; Department of Statistics and Financial Mathematics, School of Mathematics, South China University of Technology, Guangzhou 510000, Guangdong, China; Department of Statistics and Financial Mathematics, School of Mathematics, South China University of Technology, Guangzhou 510000, Guangdong, China; Department of Statistics and Financial Mathematics, School of Mathematics, South China University of Technology, Guangzhou 510000, Guangdong, China; Email: lcxia@scut.edu.cn

## Abstract

Breast cancer intrinsic subtyping is critical for precision therapy and prognostic assessment. We applied the DNA-level multi-omics classifier UGES (Unified Genetic and Epigenetic Subtyping) [1] to two independent cohorts, TCGA (n = 931) and METABRIC (n = 1134), using a hierarchical learning strategy to progressively distinguish Basal-like, HER2-enriched, Luminal A, and Luminal B subtypes. UGES demonstrated excellent overall classification performance, achieving an AUC of 0.963 on the combined test set. In the challenging task of discriminating Luminal A from Luminal B tumors, UGES reached an AUC of 0.948, substantially outperforming a single-step strategy (0.828), highlighting the benefit of the stepwise approach. Compared with PAM50, UGES reclassified 11.57% of samples, with the most notable change being 11.25% of PAM50 Luminal A samples reassigned to UGES Luminal B. Additionally, UGES improved the sensitivity for HER2-enriched subtype recognition by 28.92%. Survival analyses revealed that UGES-defined subtypes provided stronger prognostic discrimination, with significant survival differences observed across most subtype pairs, particularly for HER2 compared with other subtypes. These findings demonstrate that UGES offers a robust and clinically relevant framework for breast cancer subtyping across independent cohorts, providing a reliable tool for real-world data analysis and clinical decision-making in precision oncology.

**References:**

[1] Chang X., Xie J., Duan H., Li K., Liu X., Xiong Y., Bai X., Ning K., Xia L.C. ‘A unified genetic and epigenetic model to predict breast cancer intrinsic subtypes using large DNA-level multi-omics data and hierarchical learning.’ IEEE Transactions on Computational Biology and Bioinformatics 2025; early access. Doi:10.1109/TCBBIO.2025.3613591.

